# Drug response analysis for scaffold-free cardiac constructs fabricated using bio-3D printer

**DOI:** 10.1038/s41598-020-65681-y

**Published:** 2020-06-02

**Authors:** Kenichi Arai, Daiki Murata, Shoko Takao, Anna Nakamura, Manabu Itoh, Takahiro Kitsuka, Koichi Nakayama

**Affiliations:** 1 Center for Regenerative Medicine Research, Faculty of Medicine, Saga University, Saga, Japan; 2 Department of Thoracic and Cardiovascular Surgery, Faculty of Medicine, Saga University, Saga, Japan

**Keywords:** Medical research, Biomedical engineering, Drug safety, Pharmacology, Toxicology, Cardiac regeneration

## Abstract

Cardiac constructs fabricated using human induced pluripotent stem cells-derived cardiomyocytes (iPSCs-CMs) are useful for evaluating the cardiotoxicity of and cardiac response to new drugs. Previously, we fabricated scaffold-free three-dimensional (3D) tubular cardiac constructs using a bio-3D printer, which can load cardiac spheroids onto a needle array. In this study, we developed a method to measure the contractile force and to evaluate the drug response in cardiac constructs. Specifically, we measured the movement of the needle tip upon contraction of the cardiac constructs on the needle array. The contractile force and beating rate of the cardiac constructs were evaluated by analysing changes in the movement of the needle tip. To evaluate the drug response, contractile properties were measured following treatment with isoproterenol, propranolol, or blebbistatin, in which the movement of the needle tip was increased following isoproterenol treatment, but was decreased following propranolol or blebbistain, treatments. To evaluate cardiotoxicity, contraction and cell viability of the cardiac constructs were measured following doxorubicin treatment. Cell viability was found to decrease with decreasing movement of the needle tip following doxorubicin treatment. Collectively, our results show that this method can aid in evaluating the contractile force of cardiac constructs.

## Introduction

New drug development requires animal testing and clinical studies to demonstrate their safety and therapeutic efficacy. Drug development is relatively time-consuming and costly and has often been discontinued due to differences in the drug response between animals and humans^1–3^. In addition, drug development is often stopped due to potential side effects on the heart. Thus, understanding the drug response of the heart is essential for new drug development. Drug-induced cardiotoxicity resulting in serious adverse events such as myocardial infarction, myocardium necrosis, and lethal arrhythmia is a major reason to abort drug development before clinical application^4,5^. Thus, alternative approaches to animal testing are highly desired for new drug development.

In preclinical studies to develop new drugs, many researchers have used the two-dimensional (2D) monolayer cardiomyocyte culture as a conventional method to evaluate drug response and cardiotoxicity^6–9^. This method can measure the field potential duration and action potential of each cardiomyocyte using multi-electrode arrays and the contraction of cardiomyocytes using movie analysis software. However, in contrast to the heart *in vivo*, 2D-cultured cardiomyocytes lack high cell density and interaction between cells and the extracellular matrix. Thus, the contractile behaviour of a single cardiomyocyte differs from that of 3D cardiac tissue^10^. Therefore, *in vitro* fabrication of 3D cardiac tissue models is needed for pharmaceutical assays.

Many researchers have studied tissue engineering to fabricate 3D cardiac constructs *in vitro*^11–13^, in which scaffolds such as collagen, fibrin gel, and oriented fibres are generally used. Cardiomyocytes seeded in the scaffold demonstrate heart-specific functions, because the scaffold-based system can provide a 3D culture environment for cells. Ronaldson-Bouchard *et al*. developed 3D functional cardiac constructs using fibrin gel^14^. Although their cardiac model could partially represent the heart-specific function, cardiomyocytes in a gel cannot reproduce the high cell density condition and cell-to-cell interaction. In addition, it is possible that the materials of the cardiac constructs may interact with the screening drug. Nugraha *et al*. reported difficulties in evaluating the correct drug response of constructs, because the surface of the scaffold material (such as collagen and cellulosic gel) absorbed the hydrophilic or hydrophobic drug^15^. Thus, engineered cardiac constructs should be fabricated without a scaffold to evaluate the correct drug response of cardiac constructs.

**Figure 1:**
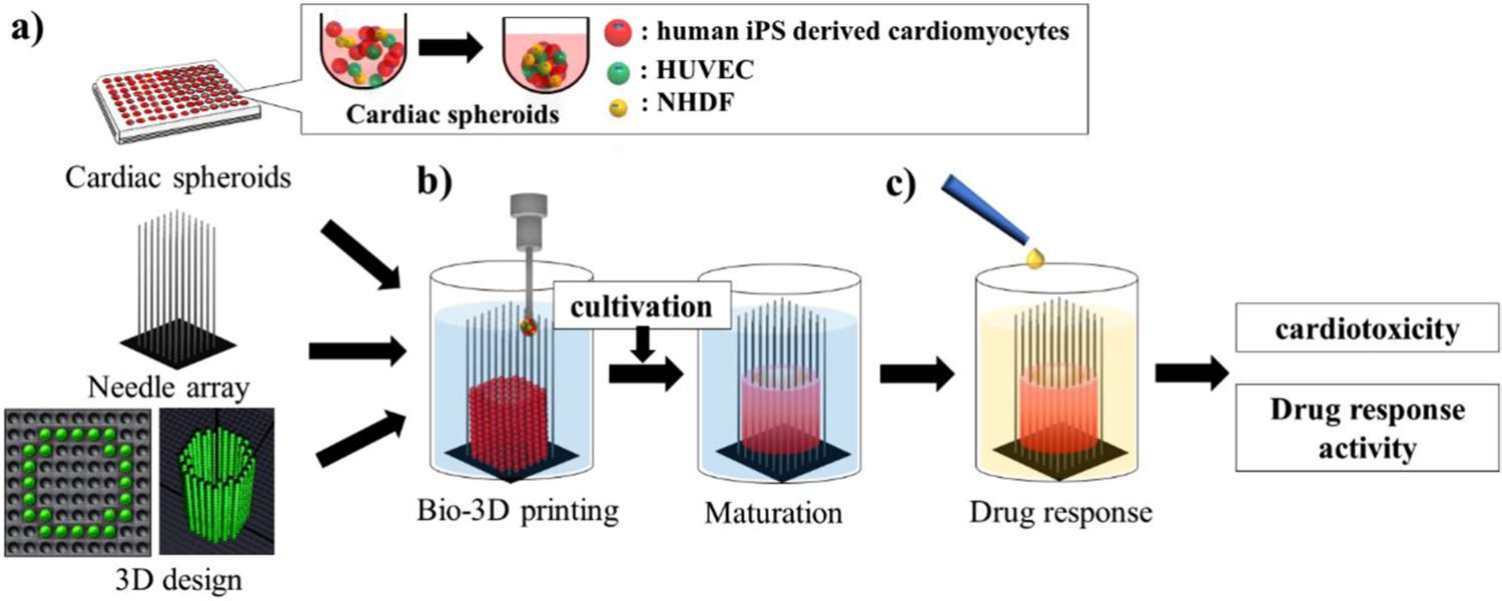
Bio-3D printing process and drug response of cardiac constructs. Cells aggregate as spheroids. The appropriate needle array and desired 3D design were selected and prepared (**a**). The spheroids were then printed onto the needle array (**b**). After cultivation, the drug response was measured using the fabricated cardiac constructs (**c**).

To overcome these challenges, we fabricated scaffold-free cardiac constructs using a bio-3D printer^22^. Cell aggregates as spheroids were printed onto a needle array according to the desired 3D design **(**Fig. 1**)**^16–23^. Our fabricated cardiac constructs were evaluated for contractility and response to electrical stimulation^22^. Therefore, we expect that the fabricated cardiac constructs can be used for clinical therapy application as well as drug response and cardiotoxicity tests. However, a method for evaluating the contractile force of these cardiac constructs has not been established yet. Here, we report a method to analyse the movement of the needle tip as an indicator of contractile force in cardiac constructs on a needle array, as well as the drug response and cardiotoxicity of cardiac constructs.

## Results

### Fabrication of tubular cardiac constructs

**Figure 2:**
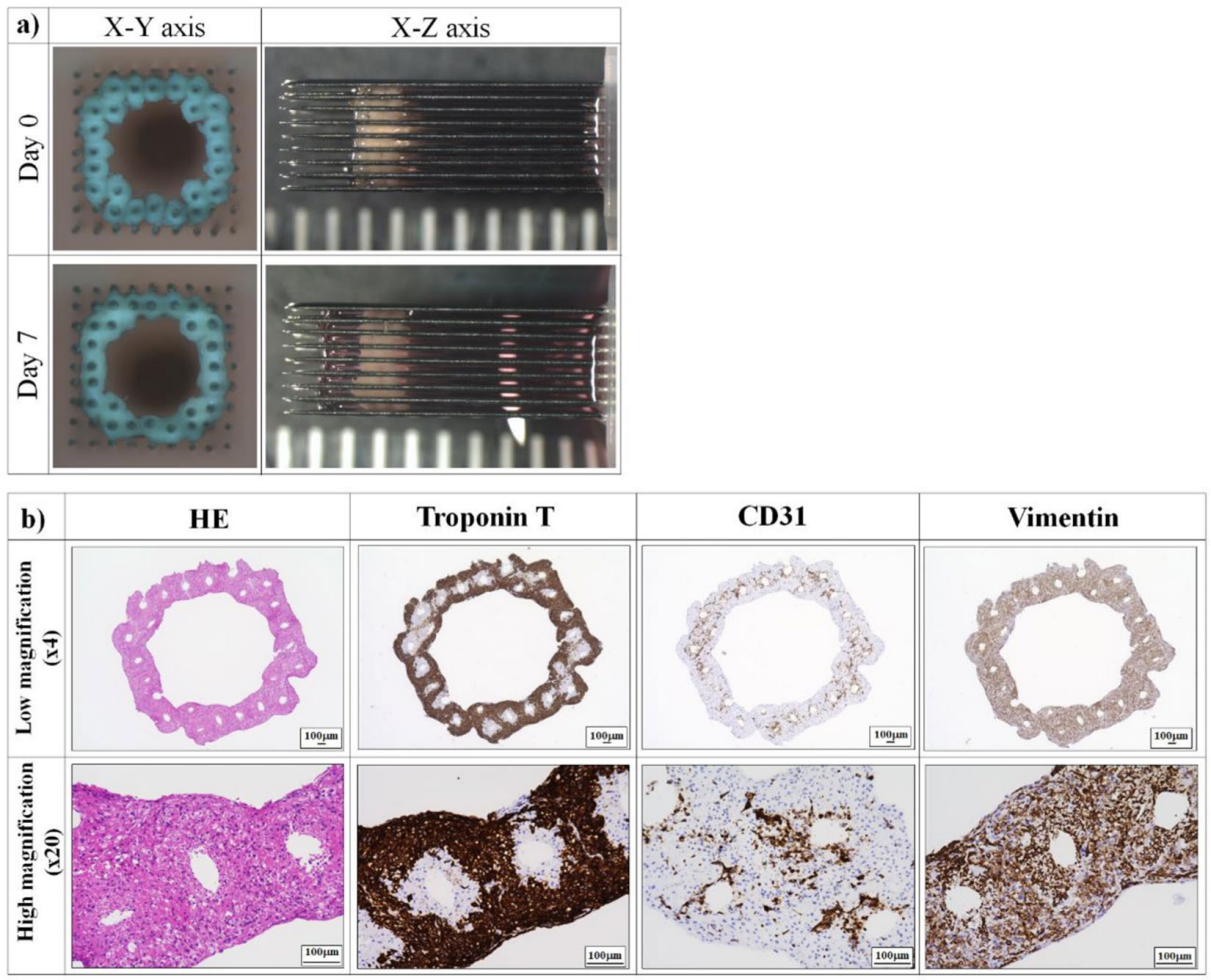
Fabrication of scaffold-free tubular cardiac constructs. (**a**) Representative images of the fabricated tubular cardiac constructs immediately following printing and culture on the needle array for 7 days. (**b**) Immunohistochemical analysis of the cardiac constructs. The sample were observed at low (4×) and high (20×) magnification. iCells were identified by troponin T staining, whereas HUVECs and NHDFs were stained with CD31 and vimentin, respectively. Scale bar = 100 *μ*m.

Although 5-day-old spheroids did not fuse with each other immediately after printing, the cardiac construct on the needle array showed spheroid fusion after an additional 7 days of culture **(**Fig. 2a**)**. The sections of cardiac constructs stained with haematoxylin and eosin (H&E) demonstrated the consistent structure and equal distribution of nuclei. Expression of Troponin T, a cardiomyocyte marker of human induced pluripotent stem cell-derived cardiomyocytes 2 (iCell2), was observed in the outer region of the constructs, whereas CD31 expression (endothelial cell marker of human umbilical vein endothelial cells (HUVECs)) was observed in the inner region. Vimentin expression (fibroblast marker of Normal human dermal fibroblasts (NHDF)) was detected throughout the construct **(**Fig. 2b**)**.

### Establishment of a contraction analysis system for the drug response of cardiac constructs

**Figure 3:**
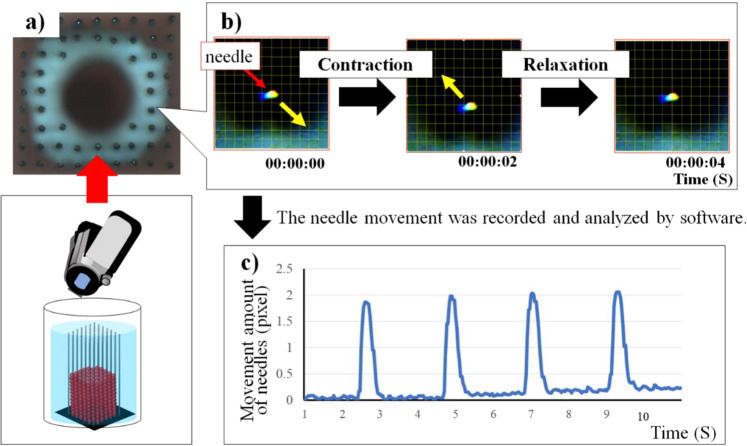
Motion analysis of the needle array movement by contraction of cardiac constructs. The cardiac constructs on the needle array were recorded (**a**), and the movement of the needle array was tracked using software (**b**). Measurement and analysis of the needle movements (**c**).

First, needle bending in relation to cardiac beats was measured to evaluate the contractile force of cardiac constructs **(**Fig. 3a**)**. The movement was recorded and analysed using laboratory-developed software **(**Fig. 3b**)**. As a result, movement of the needle tip could be easily analysed as the cardiac constructs contracted **(**Fig. 3c**)**.

**Figure 4:**
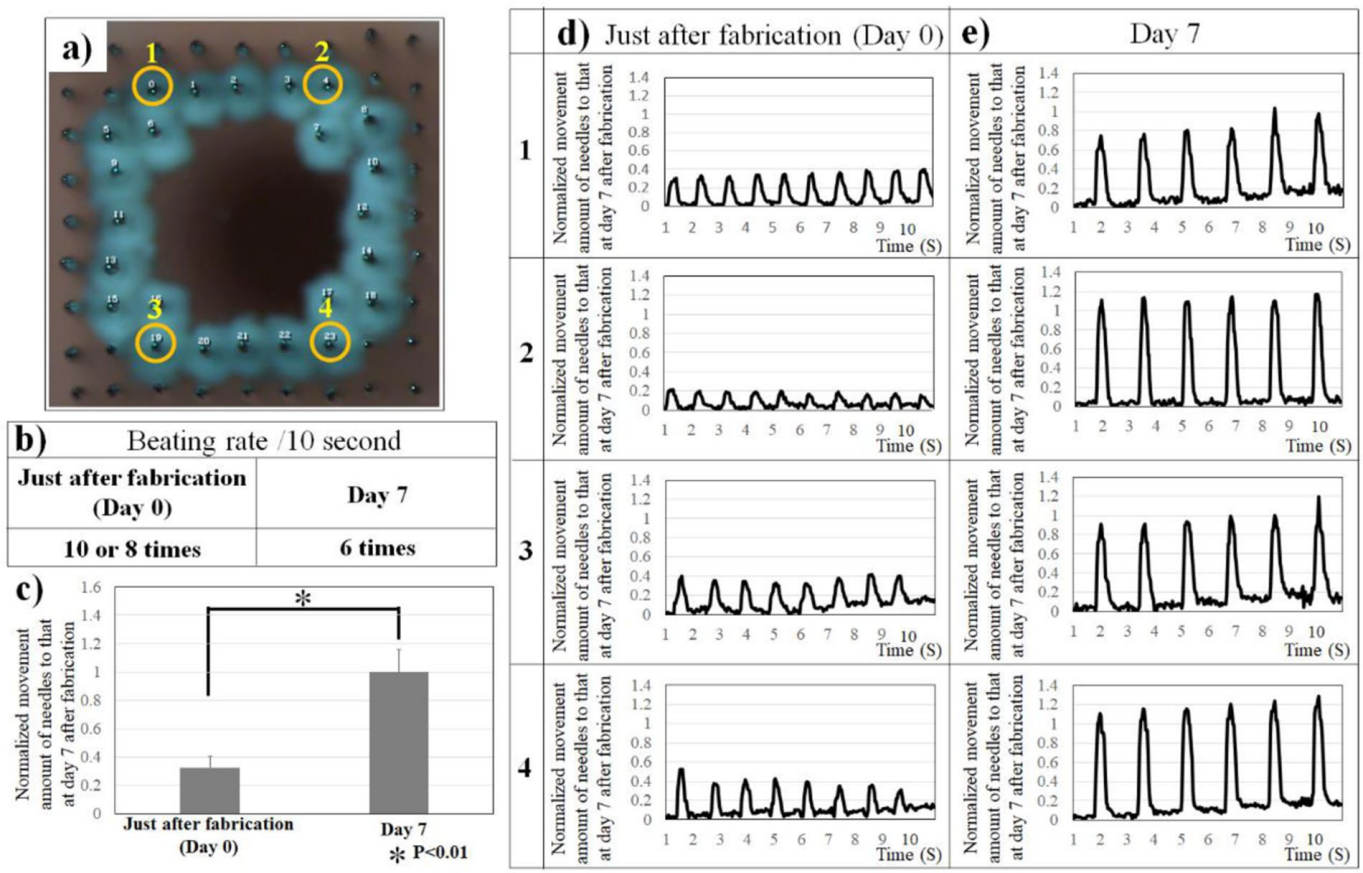
Change in the spontaneous contractile frequency and force of cardiac constructs in culture. Four points on the needle arrays were selected to evaluate contraction of the cardiac construct (**a**), and the changes in beating rate/ 10 s **(b**) and in the contractile frequency and force of cardiac constructs (**c**) were analysed at day 0 and day 7. N = 8 for each group, *P < 0.01. Error bars represent standard deviation. The movements of the needle array of the cardiac constructs at day 0 and day 7 were compared (**d**,**e**).

Next, changes in the movement of the needle tip just after fabrication of the cardiac constructs and 7 days later were measured at four points of the needle array **(**Fig. 4a**)**. The beating rate at point 1 and 2 was up to 10 beats per 10 s and 8 beats per 10 s at point 3 and 4 just after construct fabrication **(**Fig. 4b**)**. Conversely, the beating rate of the cardiac construct was 6 beats per 10 s at day 7 after construct fabrication **(**Fig. 4c**)**. Although beat synchronisation of the cardiac constructs could not be confirmed just after fabrication, changes in the movement of the needle tips increased, and beat synchronisation of the cardiac constructs was confirmed at day 7 after construct fabrication (Fig. 4d,e).

### Electrical stimulation to cardiac constructs

**Figure 5:**
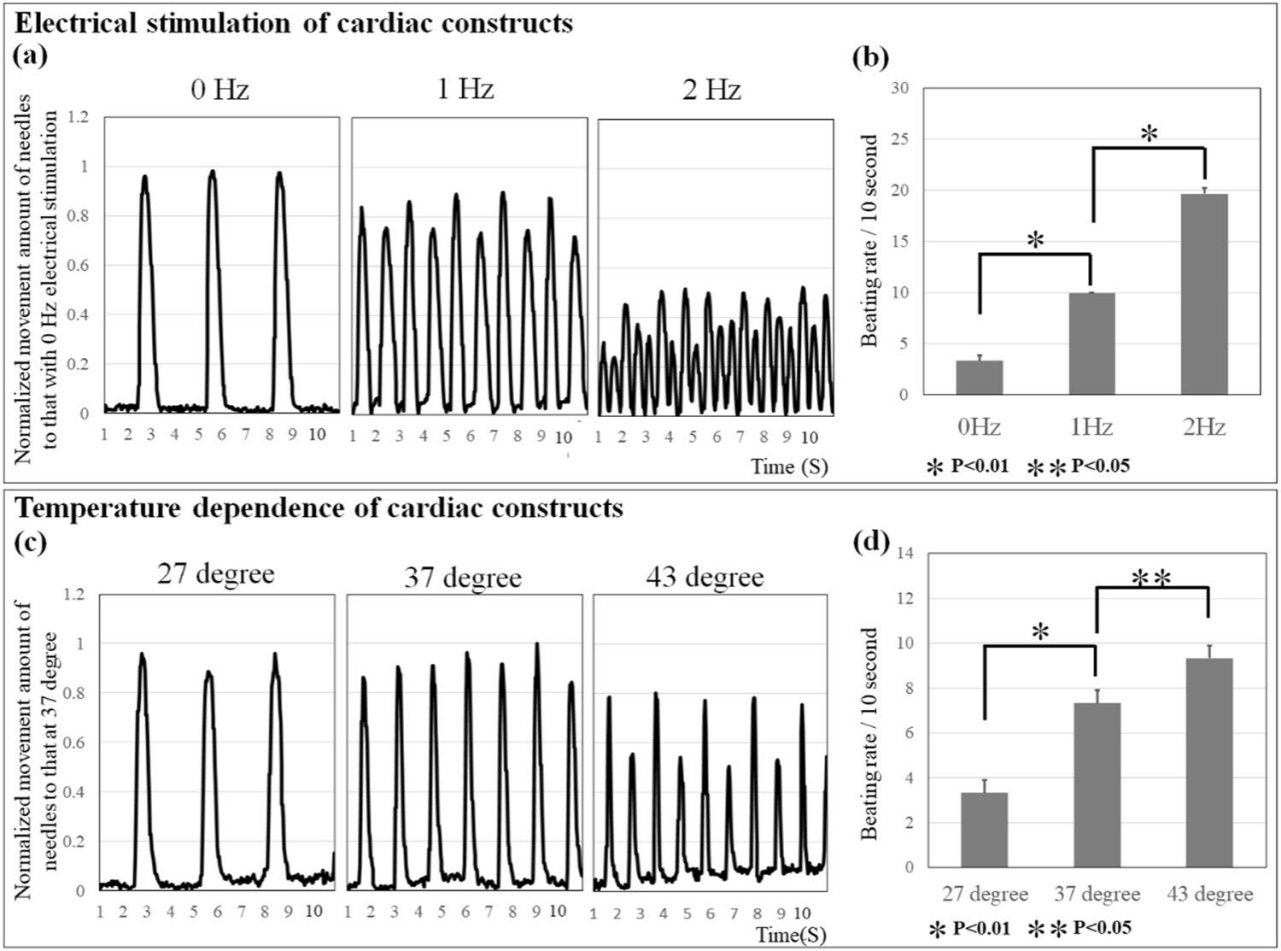
Changes in the movement of the needle tip as an indicator of contractile force and the beating rate of cardiac constructs under different conditions. Changes in the movement of the needle tip and the beating rate of cardiac constructs were confirmed by electrical stimulation (**a,b**) or temperature change (**c,d**). N = 3 for each group, *P < 0.01, **P < 0.01. Error bars represent standard deviation.

Electrical stimulation was applied to induce contraction of the cardiac constructs on the needle array **(**Supplementary Video 1**)**. As a result, spontaneous contraction of the cardiac constructs at 3 beats per 10 s increased to 10 beats per 10 s with 1 Hz electrical stimulation and to 20 beats per 10 s with 2 Hz **(**Fig. 5a,b**)**. Furthermore, changes in the movement of the needle tip decreased after 2 Hz electrical stimulation, compared to those with spontaneous contraction.

### Temperature dependence of spontaneous contraction in cardiac constructs

The beating rate of cardiac constructs was evaluated following incubation at 27 °C, 37 °C, and 43 °C **(**Fig. 5c,d and Supplementary Video 2**)**. The beating rate of cardiac constructs incubated at 37  °C was 7 beats per 10 s. Conversely, the beating rate of cardiac constructs incubated at 43 °C was 9 beats per 10 s, whereas constructs incubated at 27 °C decreased to 3 beats per 10 s. These results indicated that the beating rate of cardiac constructs was dependent on the temperature of the culture medium.

### Drug reactivity analysis of cardiac constructs

**Figure 6:**
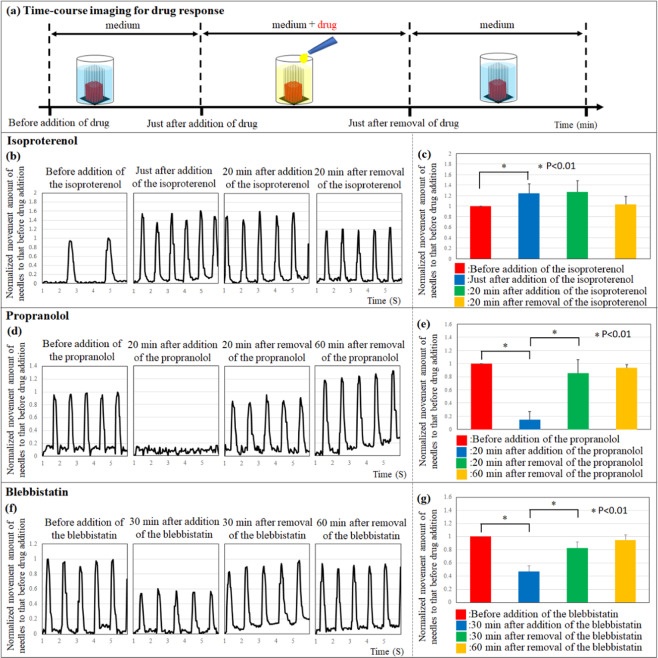
Drug reactivity of cardiac constructs. Time-course imaging for drug response (**a**). Top movement of the needle array of the cardiac constructs upon treatment with isoproterenol (**b,c**), propranolol (**d,e**), or blebbistatin (**f,g**), and after the removal of these drugs. N = 3 for each group, *P < 0.01. Error bars represent standard deviation.

We evaluated the drug response of cardiac constructs to isoproterenol, propranolol, and blebbistatin **(**Fig. 6a, Supplementary Video 3, 4, and 5**)**. The result showed that the beating rate and the change in movement of the needle tip increased following isoproterenol treatment **(**Fig. 6b,c**)**. After the cardiac constructs were incubated in medium with isoproterenol for 30 min, the culture medium was replaced with fresh medium without isoproterenol. Further, 20 min after removal of isoproterenol, the change in the movement of the needle tip gradually returned to baseline levels. Conversely, the beating rate and change in the movement of the needle tip decreased dramatically at 20 min after propranolol addition **(**Fig. 6d,e**)**. After the cardiac constructs were incubated in medium containing propranolol for 30 min, the culture medium was replaced with fresh medium without propranolol. Additionally, 60 min after propranolol removal, the change in the movement of the needle tip returned to baseline levels. Although the top movement of needles decreased at 30 min after the addition of blebbistatin, the beating rate of cardiac constructs did not change **(**Fig. 6f,g**)**. After the cardiac constructs were incubated in medium with blebbistatin for 30 min, the culture medium was replaced with fresh medium without blebbistatin. Further, 60 min after removal of blebbistatin, the change in needle tip movement returned to baseline levels.

### Cytotoxicity effect of doxorubicin on cardiac constructs

**Figure 7:**
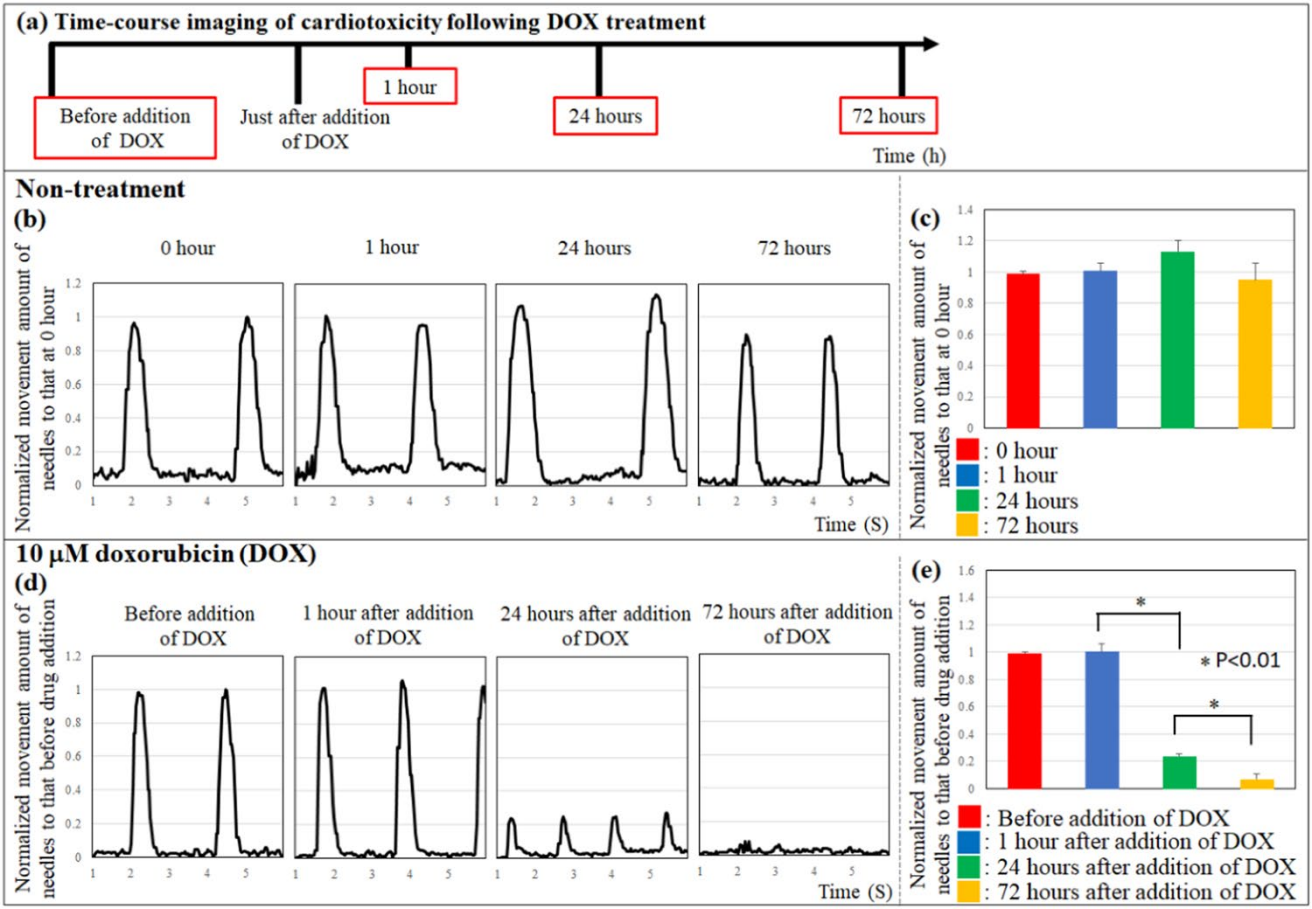
Cardiotoxicity in cardiac constructs following doxorubicin treatment. Time-course imaging of cardiotoxicity following DOX treatment (**a**). The movement of the needle array did not change in response to non-treatment (**b,c**). Conversely, the top movement of the needle array decreased in response to 10 μM doxorubicin treatment (**d,e**). N = 3 for each group, *P < 0.01. Error bars represent standard deviation.

To confirm whether the contraction analysis system of cardiac constructs could be used as an alternative to animal experiments, we evaluated the cytotoxicity effect of doxorubicin (DOX) on the cardiac constructs **(**Fig. 7a, Supplementary Video 6**)**. The beating rate and the top movement of needles of cardiac constructs cultured without DOX for 72 h did not change **(**Fig. 7b,c**)**. In contrast, although the beating rate and the needle tip movement did not change 1 h after DOX addition, the change in needle tip movement decreased dramatically 24 h following DOX addition. Furthermore, contraction of the cardiac construct completely ceased at 72 h after DOX treatment **(**Fig. 7d,e**)**.

**Figure 8:**
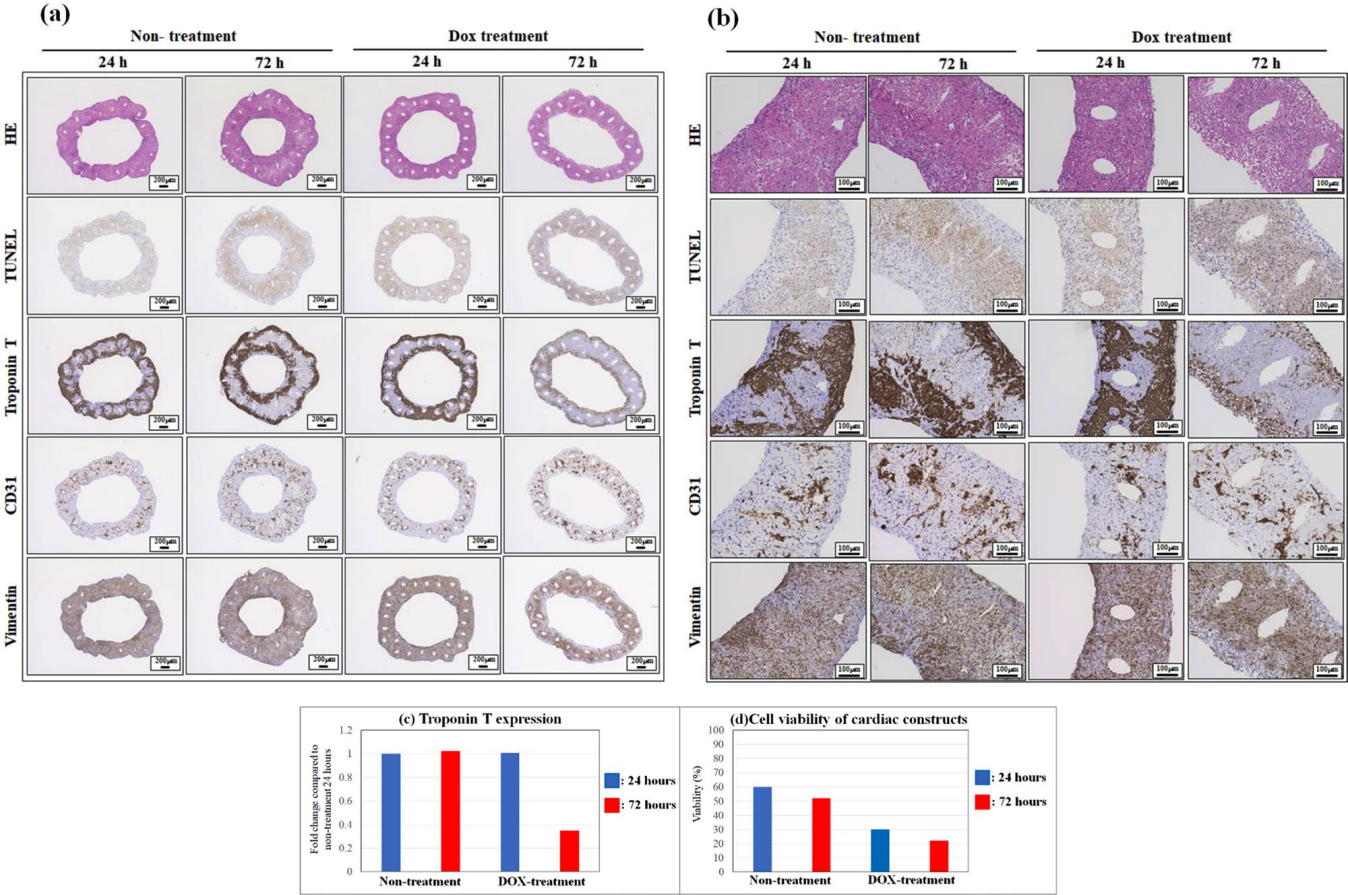
Immunohistochemical analysis of cardiac constructs following doxorubicin treatment. The samples were observed at low (4×) and high (10×) magnification (**a**,**b**). iCells were identified by troponin T staining, whereas HUVECs and NHDFs were stained with CD31 and vimentin, respectively. Troponin T expression in cardiac constructs was measured at 24 and 72 h following DOX treatment (**c**). Cell viabilities in cardiac constructs were measured by counting the number of dead cells (**d**). Troponin-T expression and cell viability in cardiac constructs were analysed using Image analysis software BZ-H3A.

Expression of troponin T in DOX-treated cardiac constructs for 72 h was decreased compared to that in untreated constructs **(**Fig. 8c**)**. Conversely, the expression of CD31 and vimentin in cardiac constructs did not change with DOX treatment for 72 h **(**Fig. 8a,b**)**. In addition, the number of TUNEL stain-positive cells was increased in the cardiac construct treated with DOX for 72 h **(**Fig. 8d**)**. These results indicate that the contraction analysis system can evaluate the cardiotoxicity effect on cardiac constructs following drug treatment.

## Discussion

A system to evaluate the 3D contractile force of cardiac constructs is important for new drug development. Although several research groups have already reported the fabrication of scaffold-based cardiac constructs, these scaffold-based cardiac constructs cannot completely reproduce the drug response of the heart *in vivo* due to interactions between the drug and scaffold materials^15^. Although scaffold-free cardiac constructs such as patches and spheroids have been fabricated, a system for evaluating the contractile force of 3D engineered scaffold-free cardiac constructs has not been reported. In this study, we established an analysis system that can measure changes in the movement of the needle tip as an indicator of contractile force in cardiac constructs on a needle array, related to the drug response.

First, the fabricated tubular cardiac constructs were cultured in a bioreactor to promote spheroid fusion on the cardiac constructs by perfusing the culture medium. Cardiomyocytes in the cardiac constructs were rearranged to the outer surface of the constructs after cultivation. Cardiomyocyte rearrangement may have occurred by recognising the outer surface of the cardiac constructs as the blood vessel side, because the cardiomyocytes require sufficient nutrition and oxygen^24^. After the drug is absorbed *in vivo*, it reaches cardiomyocytes in the heart by flowing through the bloodstream in the blood vessel. We believe that our fabricated cardiac constructs can represent the drug response because the cardiomyocytes recognise the surface of the construct, which is in contact with the culture medium, as the blood vessel. Taken together, the localisation of cardiomyocytes in constructs is an important behaviour to evaluate the drug effects using this contraction analysis system.

Although several research groups have developed 3D cardiac constructs, it is difficult to evaluate the contractile force of cardiac constructs in three dimensions^25–27^. Although they have evaluated the contraction and beating rate by analysing changes in the construct area during contraction of engineered cardiac constructs, this method cannot evaluate the contraction of 3D cardiac constructs. In this study, to solve this problem, we focused on movement of the needle tip during contraction of the cardiac constructs. When the cardiac construct contracts, the contractile force of the cardiac construct is directly transmitted to the needle array. However, the movement of the needle tip could not be converted into the contractile force of cardiac constructs, because the length of the needle tip was not uniform at the micro-level. Further, we could not obtain the properties (material, Young’s modulus, length) needed to convert the movement of the needle tip into the contractile force of the constructs. To investigate contraction of the cardiac construct, we confirmed the optimised culture conditions of the cardiac constructs. Because printed cardiac spheroids in the cardiac constructs did not fuse with each other and mature immediately following fabrication, the movement at four points of the needle array was very small and the beating of cardiac constructs was not synchronised. Several researchers have reported that the phenotype of induced pluripotent stem cells-derived cardiomyocytes (iPSCs-CMs) is not mature, compared to adult cardiomyocytes^28,29^. For example, the contractile force of foetal cardiomyocytes and iPSCs-CMs is weaker than that of adult cardiomyocytes^30–32^. Further, the beating rate of foetal cardiomyocytes and iPSCs-CMs is faster than that of adult cardiomyocytes. To overcome these challenges, it has been reported that the maturation of foetal cardiomyocytes and iPSCs-CMs can be promoted by a 3D culture environment, electrical stimulation, physical stimulation, and growth factors^33–41^. Our results indicated that the maturation of iPSCs-CMs in cardiac constructs was stimulated because their beating rate was decreased and their change in movement was increased at 7 days after fabrication. Thus, we used cardiac constructs cultured on the needle array for 7 days to evaluate drug responses.

To confirm whether the fabricated cardiac constructs were representative of the contraction behaviour of the heart *in vivo*, we examined the electrical stimulation response of the cardiac constructs. In general, it is known that the cardiac output of the human heart decreases when its beating rate increases to more than 150 times/min, because full relaxation of the heart is prevented. Therefore, an accurate assessment of the effect of contractile force of the cardiac construct by an increased beating rate using the contraction analysis system is needed. To modulate the beating rate of cardiac constructs, electrical stimulation was applied. During the spontaneous contraction condition, the beating rate of cardiac constructs was 3 beats per 10 s. When 1 or 2 Hz-paced electrical stimulation was applied to cardiac constructs, the beating rate increased. However, 2 Hz paced electrical stimulation prevented full relaxation of the cardiac constructs and showed a decrease in the top movement of the needle. Stevens *et al*. also demonstrated that scaffold-free cardiac patches showed a decrease in contractile force at 2 Hz or 3 Hz paced electrical stimulation^26^. Thus, our results indicate that this contraction analysis system can reproduce the contraction of the human heart.

The temperature condition of the cardiac construct must remain constant during the drug response test, because the beating rate of the heart is temperature dependent. Jonsson *et al*. have reported that temperature changes can affect the beating rate of iPSCs-CMs cultured in monolayers^42^. The beating rate of our cardiac constructs decreased at lower temperature (27 °C). Conversely, the cardiac constructs showed an increased beating rate at physiological and high temperatures (37 °C, 43 °C), confirming the temperature control of cardiac constructs as a very important parameter for evaluating drug responses.

Finally, we applied four drugs (isoproterenol, propranolol, blebbistatin, and doxorubicin) to validate the contraction analysis system for drug response and cardiotoxicity monitoring. We selected well-known drugs, isoproterenol and propranolol, because of their ability to change the beating rate and contractile force of cardiomyocytes. In general, isoproterenol is known to increase the contractile force and beating rate of cardiomyocytes upon binding to *β*-receptors on the cell membrane. In contrast, propranolol is known to decrease the contractile force and beating rate by blocking the *β*-receptors. In this study, we detected changes in the needle tip movement as contractile force and those in the beating rate after addition isoproterenol or propranolol. Further, the movement of the needle tip as an indicator of contractile force returned to the initial values after removal of the drugs. These results indicated that the cardiac construct in the contraction analysis system showed a reversible drug response. Beauchamp *et al*. previously reported about the isoproterenol reactivity of hiPS-CMs spheroids by analysing the changes in the spheroid area upon contraction^43^. Although the beating rate of hiPS-CMs spheroids increased after the addition of isoproterenol, the area of spheroid motion did not change. The authors measured the change in spheroid area as contractile force using video recording. However, we presume that the 3D contractile force of hiPS-CMs spheroids was not measured, because the change in the area of the 2D cross section can only be obtained from video recording of spheroids. In this contraction analysis system, we evaluated the change in the movement of the needle tip as an indicator of contractile force in the cardiac construct. Blebbistatin is a cell-permeable inhibitor of heart muscle myosin and non-muscle myosin II ATPase. Although changes in the needle tip movement decreased after addition of blebbistatin, the beating rate of the cardiac constructs did not change. In general, blebbistatin treatment in cardiomyocytes decreases contractile force, but the field potential and beating rate of cardiomyocytes are not affected^44^. Therefore, using this contraction analysis system, the drug response in cardiac constructs can be accurately evaluated by measuring the contractile force in two dimensions.

The most important challenge in drug development is cardiotoxicity to cardiomyocytes. In this study, we confirmed that the cardiotoxicity of constructs could be evaluated using this drug reactivity system. Doxorubicin (DOX) was used as model drug to assess cardiotoxicity in the contraction analysis system. DOX is known as one of the antineoplastic agents with cardiotoxic effects, which inhibits Ca ion handling and mitochondrial function. Shimauchi *et al*. reported that reactive oxygen species levels in cardiomyocytes of the heart increased with DOX treatment, and cardiotoxicity and apoptosis in cardiomyocytes were induced^45^. Several research groups evaluated the DOX-induced cardiotoxicity in cardiomyocytes *in vitro*. Jahnke *et al*. reported that the contractile force and beating rate of cardiac spheroids decreased with DOX treatment^46^. Although the contractile force of cardiac spheroids did not change 1 h after addition of 0.01–10 μM DOX, a decrease in cell viability could be observed 48 h after DOX additions, even at the lowest concentration of 0.01 μM. Our results were consistent with those of the previous study, and the beating rate and the movement of the needle tip as an indicator of contractile force in the cardiac constructs did not change at 1 h following the addition of DOX. Additionally, the change in needle tip movement as contractile force decreased 24 h after DOX addition, and contraction of the cardiac construct stopped 72 h after DOX treatment. Further, the cell viability and troponin T expression of cells in the cardiac constructs decreased with DOX. Taken together, these results indicate that the cardiotoxicity of cardiac constructs can be evaluated using the contraction analysis system and histological analysis (cell viability and troponin T).

Although the contraction analysis system can work properly and effectively for drug response and cardiotoxicity, there are several limitations with the system. First, the top movement of the needle could not be converted as contractile force of the cardiac constructs. To convert the change in needle tip movement as contractile force, properties, such as material, Young’s modulus, diameter, and length, of needle are needed. We could not calculate the contractile force of the construct, because the length of needle array was not uniform at the micro-level. In future, we will evaluate the contractile force accurately by improving the length of the needle array. Second, we used the frame rate (30 frames per s) of our video camera to evaluate contraction of the cardiac constructs. Conversely, Takeda *et al*. reported that a video camera recording at 150 frames per s can measure the contraction velocity of cardiomyocytes as well as the relaxation velocity and contraction-relaxation velocity^47^. Thus, in the future, we aim to perform contraction analysis of cardiac constructs using a video camera with a high frame rate.

In conclusion, we established the contraction analysis system of cardiac constructs for drug response, using a bio-3D printer and needle array. This system can evaluate contractile force of the cardiac constructs by analysing changes in the movement of the needle tip. We confirmed the contraction analysis system efficiency by observing different drug responses and cardiotoxicity in our cardiac construct, which was dependent on the type of applied drug. This analysis method can be used for new drug development, due to its high drug response predictability *in vitro*. We believe that accuracy of this contraction analysis system will increase in the future due to advances in camera technology and the quality of the needle array.

## Materials and Methods

### Cell culture and cardiac spheroids formation

Normal human dermal fibroblasts (NHDF) and Human umbilical vein endothelial cells (HUVECs) were purchased from Lonza, Inc. (Walkersville, MD, USA). Cells were cultured in FBM-2 and EBM-2, respectively. Human induced pluripotent stem cells-derived cardiomyocytes (iCells2) were purchased form Cellular Dynamics International Japan Co., Ltd (CDI, Tokyo, Japan). Cryopreserved iCell2 cells were thawed for 4 min in a water bath at 37 °C, diluted in iCell Plating Medium (CDI, Tokyo, Japan), and used for spheroid formation. The cell suspension solution composed of iCell2 (50%), HUVECs (25%), and NHDF (25%) was seeded into ultra-low attachment 96 U-well plates (SUMITOMO BAKELITE, Tokyo, Japan) to form cardiac spheroids containing a total of 35,000 cells per well.

### Fabrication of scaffold-free cardiac constructs using a Bio-3D printer

Cardiac constructs fabrication method has been previously reported^22^. The cardiac constructs were printed using 5-day-old cardiac spheroids and cultured on the needle arrays for an additional 7 days before use in the drug response experiment. To evaluate drug response of cardiac constructs reproducibly, we fabricated the three layers of tubular cardiac constructs using spheroids of the same size.

### Histological and immunohistochemical analysis

The cardiac constructs were stained and observed according to our previously published paper^22^. The samples were fixed in 10% formalin neutral buffer solution for 48 h at 4 °C and embedded in paraffin, and cut into 5 μm sections. The sections were used for haematoxylin and eosin (H&E) staining or immunohistochemistry. To stain cardiomyocyte, endothelial cells, and fibroblasts in cardiac constructs, primary antibodies against troponin T (dilution 1:100; MS-295-P0, Thermo Fisher Scientific, Inc., Waltham, MA, USA), CD31 (dilution 1:75; NCL-CD31–1A10, Leica Biosystems, Heidelberger, Germany), and vimentin (dilution 1:300; Biocare Medical, Pacheco, CA, USA) were used. To measure cell viability of the constructs, the sections were stained using the *in situ* cell death detection kit (Roche Applied Science, Burgess Hill, UK) according to the manufacturer’s instructions. The sections were observed using a BZ-X700 microscope (Keyence, Osaka, Japan).

### Contraction analysis system of cardiac construct

To analyse contraction force and beating rate of the cardiac construct located on the needle array, movement of the needles was recorded using a digital camera (Leica MC120 HD, Leica Microsystems Inc. Buffalo Grove, IL, USA) mounted on a stereoscopic microscope SZX7 (Olympus, Tokyo, Japan). Motion was analysed using a laboratory-developed software program that could recognise the top of needles in the array and track the distance of movement. The laboratory-developed software program (version 1.11) could be downloaded from https://github.com/Nlabs-7652/Bending_Analyzer/releases/. During calculation, the movement of the needle tip after the drug addition was normalized to that before drug addition (baseline level).

### Electrical stimulation

The electrical stimulation device has been previously reported^22^. A PSW 80–13.5 was used as the electric power supply (Good Will Instrument Co., Ltd, New Taipei City, Taiwan). The cardiac constructs on the needle array were transferred to the chamber and stimulated with bipolar electrical pulses of 20 V and 1 Hz or 2 Hz for 10 ms (repeated every 990 ms or 490 ms, respectively).

### Temperature dependence of contraction characteristics in cardiac constructs

To evaluate the temperature dependence of cardiac constructs, the effect of temperature on contractile force and beating rate was assessed by analysing contraction of cardiac constructs incubated for 30 min in three different temperature conditions at 27 °C, 37 °C, and 43 °C.

### Drug reactivity analysis

To evaluate the drug response of the cardiac constructs, isoproterenol (Sigma-Aldrich, St Louis, MO, USA), propranolol (Sigma-Aldrich), and blebbistatin (Wako Pure Chemical Industries, Ltd, Osaka, Japan) were used. Each drug was added to culture medium (iCell maintained medium: EGM-2: FBM = 1:1:1). The final concentration of isoproterenol, propranolol, and blebbistatin was 1 μM, 5 μM, and 500 nM, respectively. The cardiac constructs were incubated for 30 min in culture medium with the investigated drugs. After incubation, the culture medium was removed and replaced with fresh medium without drug and cultured for an additional 30 min. Cardiac construct contraction was recorded and analysed at indicated time points.

### Cardiotoxicity of doxorubicin

Briefly, 10 µM doxorubicin (DOX) was administered to the cardiac constructs for 72 h in culture medium. Contraction of the cardiac constructs was recorded at 1, 24, and 72 h. Cardiac constructs were fixed at 24 and 72 h and stained with Troponin-T, CD31, and vimentin. To assess cell spatial distribution within the cardiac constructs, sections were also stained with H&E. Troponin-T expression and cell viability in cardiac constructs before and after DOX addition were analysed using Image analysis software BZ-X series (version: BZ-H3A, https://www.keyence.com/products/microscope/fluorescence-microscope/bz-x700/models/bz-h3ae/) (Keyence, Osaka, Japan).

### Statistical analysis

All numerical data are presented as mean ± standard deviation (SD). The values represent the means ± SD from three independent experiments. Comparisons between the two groups were analyzed by a Student’s *t*-test using Microsoft Office Excel (Microsoft, Redmond, WA). A P value < 0.01, or <0.05 indicates statistically significant differences.

## Supplementary information

Supplementary Video Information

Supplementary Video 1

Supplementary Video 2

Supplementary Video 3

Supplementary Video 4

Supplementary Video 5

Supplementary Video 6

## Data Availability

All data generated or analysed during this study are included in this published article (and its additional information files).
