# Supplementary material for: Drug response analysis for scaffold-free cardiac constructs fabricated using bio-3D printer
Source: Sci Rep. 2020 Jun 2;10:8972. doi: 10.1038/s41598-020-65681-y (PMC7265390; doi:10.1038/s41598-020-65681-y)
Supplement: Supplementary file 1 — Supplementary Video Information [file 41598_2020_65681_MOESM1_ESM.pdf]

Supplementary Information

Drug response analysis for scaffold-free cardiac constructs fabricated using bio-3D printer

Kenichi Arai[1]*, Daiki Murata[1], Shoko Takao[1], Anna Nakamura[1], Manabu Itoh[2], Takahiro Kitsuka[2], Koichi Nakayama[1]*

[1] Center for Regenerative Medicine Research, Faculty of Medicine, Saga University, Saga, Japan

[2]Department of Thoracic and Cardiovascular Surgery, Faculty of Medicine, Saga University, Saga, Japan

E-mail addresses: arai0472@cc.saga-u.ac.jp (Kenichi Arai), daiki_net_official@yahoo.co.jp (Daiki Murata), shoko_takao@nakayama-labs.com (Shoko Takao), anna.nakamura@nakayama-labs.com (Anna Nakamura), itomana@cc.saga-u.ac.jp (Manabu Itoh), sr1123@cc.saga-u.ac.jp (Takahiro Kitsuka), nakayama@me.saga-u.ac.jp (Koichi Nakayama)

*Corresponding authors:

Kenichi Arai and Koichi Nakayama

Department of Regenerative Medicine and Biomedical Engineering, Faculty of Medicine, Saga University

1 Honjo-machi, Saga, Japan

E-mail addresses: arai0472@cc.saga-u.ac.jp (Kenichi Arai), nakayama@me.saga-u.ac.jp (Koichi Nakayama)

Supplementary Video

Supplementary Video 1. Electrical stimulation of cardiac constructs onto needle arrays.
This movie shows the effects of electrical stimulation (1 Hz and 2 Hz) in the fabricated tubular cardiac constructs.

Supplementary Video 2. Changes in contractile force and beating rate of cardiac constructs under different temperature conditions.
This movie shows the effect of different temperature conditions (27°C, 37°C, and 43°C) in the fabricated cardiac construct on the needle array.

Supplementary Video 3. Temporal changes in beating rate and needle movement by isoproterenol treatment.
Time-course imaging for drug response. Top movement of the needle array of the cardiac constructs upon treatment with isoproterenol and after removal of these drugs.

Supplementary Video 4. Temporal changes in beating rate and needle movement by propranolol treatment.
Time-course imaging for drug response. Top movement of the needle array of the cardiac constructs upon treatment with propranolol and after removal of these drugs.

Supplementary Video 5. Temporal changes in beating rate and needle movement by blebbistatin treatment.
Time-course imaging for drug response. Top movement of the needle array of the cardiac constructs upon treatment with blebbistatin and after removal of these drugs.

Supplementary Video 6. Temporal changes in beating rate and needle movement by doxorubicin treatment.
Time-course imaging of cardiotoxicity following DOX treatment. The movement of the needle array of the cardiac constructs upon treatment with doxorubicin for 1 hour, 24 hours, 72 hours.
